# Overexpression of *HvIcy6* in Barley Enhances Resistance against *Tetranychus urticae* and Entails Partial Transcriptomic Reprogramming

**DOI:** 10.3390/ijms19030697

**Published:** 2018-03-01

**Authors:** M. Estrella Santamaria, Mercedes Diaz-Mendoza, David Perez-Herguedas, Goetz Hensel, Jochen Kumlehn, Isabel Diaz, Manuel Martinez

**Affiliations:** 1 Centro de Biotecnologia y Genomica de Plantas, Instituto Nacional de Investigacion y Tecnología Agraria y Alimentaria (INIA), Campus Montegancedo, Universidad Politecnica de Madrid (UPM), 28223 Pozuelo de Alarcon, 28040 Madrid, Spain; me.santamaria@upm.es (M.E.S.); mercedes.diaz.mendoza@upm.es (M.D.-M.); d.perezh@alumnos.upm.es (D.P.-H.); i.diaz@upm.es (I.D.); 2 Departamento de Biotecnologia-Biologia Vegetal, Escuela Tecnica Superior de Ingenieria Agronomica, Alimentaria y de Biosistemas, UPM, 28040 Madrid, Spain; 3 Plant Reproductive Biology, Leibniz Institute of Plant Genetics and Crop Plant Research (IPK) Gatersleben, Corrensstrasse 3, 06466 Seeland, Germany; hensel@ipk-gatersleben.de (G.H.); kumlehn@ipk-gatersleben.de (J.K.)

**Keywords:** barley, cystatin, plant defense, transcriptomics, transgenic, *Tetranychus urticae*

## Abstract

Cystatins have been largely used for pest control against phytophagous species. However, cystatins have not been commonly overexpressed in its cognate plant species to test their pesticide capacity. Since the inhibitory role of barley HvCPI-6 cystatin against the phytophagous mite *Tetranychus urticae* has been previously demonstrated, the purpose of our study was to determine if barley transgenic lines overexpressing its own *HvIcy6* gene were more resistant against this phytophagous infestation. Besides, a transcriptomic analysis was done to find differential expressed genes among wild-type and transformed barley plants. Barley plants overexpressing *HvIcy6* cystatin gene remained less susceptible to *T. urticae* attack when compared to wild-type plants, with a significant lesser foliar damaged area and a lower presence of the mite. Transcriptomic analysis revealed a certain reprogramming of cellular metabolism and a lower expression of several genes related to photosynthetic activity. Therefore, although caution should be taken to discard potential deleterious pleiotropic effects, cystatins may be used as transgenes with impact on agricultural crops by conferring enhanced levels of resistance to phytophagous pests.

## 1. Introduction

Plant protease inhibitors are small proteins with a low molecular weight. Their role in defense resides in their capacity to inhibit heterologous proteinases from herbivorous and pathogens through a reversible tight binding reaction [1]. Therefore, inhibition of digestive herbivore gut proteases is achieved affecting their performance and reducing the plant palatability. Protease inhibitors are one of the most studied inducible plant defenses, having been largely used as effective defense molecules against different pests and pathogens [2,3,4]. Within this group of inhibitors, phytocystatins (PhyCys) superfamily is one of the most important. They are present in monocotyledons and dicotyledons including important crops such as barley, rice, maize, tomato or the model plant *Arabidopsis thaliana* [5]. Their activity, mainly inhibiting cysteine proteases (CysProt) of the C1A papain family, has been associated with both endogenous physiological processes and biotic/abiotic stress responses [6]. There are some examples of recombinant purified PhyCys with demonstrated capability to inhibit the activity of digestive proteases from insects and acari in vitro (reviewed in [7]). Likewise, in experiments using artificial diets and in multiple bioassays on plants stably transformed with PhyCys genes, alterations in digestive proteolytic profiles have been reported (reviewed in [7]). 

In barley, 13 cystatins (HvCPI-1 to HvCPI-13) have been previously identified and characterized [8,9]. They participate in endogenous plant processes and also have a role in defense and response to biotic and abiotic stresses. Their defense function against herbivores has been inferred from their capability to inhibit the activity of digestive proteases from insects and acari in in vitro experiments, using artificial diets and in bioassays on plants stably transformed [10,11,12,13]. Firstly, potato plants were transformed with a variant of HvCPI-1 with improved inhibitory properties. A decrease in growth was observed in larvae of the coleopteran *Leptinotarsa decemlineata* after feeding on these plants. Later, in vitro assays showed that HvCPI-6 had the strongest inhibitory properties against both cathepsin-L- and -B-like protease activities from phytophagous insects and acari [7]. Its protective efficiency was confirmed using artificial diets and after being expressed in transgenic plants. When supplied in artificial diets, HvCPI-6 was toxic to the aphid *Acyrtosyphon pisum*. The effects of HvCPI-6 ingestion on *A. pisum* were correlated with a decrease of cathepsin-B- and -L-like protease activities [11]. When Arabidopsis plants expressing HvCPI-6 were tested against the aphid *Myzus persicae* a decrease of the aphid reproductive rate and an increase in the length of its developmental cycle were observed [11]. Similar results were obtained with the two-spotted spider mite *Tetranychus urticae* in bioassays performed in both Arabidopsis and maize transgenic lines expressing the *HvIcy6* gene. Feeding experiments on maize transgenic lines impaired mite development and reproductive performance. Besides, a significant reduction of CysProt activities in the spider mite was observed [12]. Likewise, Arabidopsis transformed plants exhibited a lower damaged leaf area in comparison to non-transformed controls together a significant increase in mites’ mortality. These effects were more remarkable when *T. urticae* fed on double transgenic Arabidopsis plants expressing HvCPI-6 and the barley trypsin inhibitor CMe [13].

Despite their potential toxic properties, cystatins have not been commonly overexpressed in its cognate plant species to test their pesticide capacity. To date, the only analysis has been reported in sugarcane. In this study, transgenic sugarcane plants overexpressing sugarcane CysProt inhibitor 1 (CaneCPI-1) were used in feeding bioassays with the weevil *Sphenophorus levis* [14]. A reduction of the average weight was observed in larvae feeding on transgenic plants. Besides, transformed sugarcane showed less damage than non-transformed plants. On the other hand, the deterrent effect of plants overexpressing a cystatin has been directly attributed to its capacity of inhibiting insect and acari digestive proteases, and no transcriptomic analysis has been done among transgenic and non-transgenic plants to check potential collateral changes in gene expression that could affect the defense mechanisms of the plant.

Since the deterrent role of HvCPI-6 cystatin against *T. urticae* has been previously demonstrated, the purpose of our study was to determine if barley transgenic lines overexpressing its own *HvIcy6* gene were more resistant against this phytophagous mite. Besides, a transcriptomic analysis has been done to find differential expressed genes among wild-type and transformed barley plants. The potential use of transgenic barley to confer protection against phytophagous arthropods is evaluated and discussed.

## 2. Results

### 2.1. Expression of *HvIcy6* Is Altered in Barley Leaves during the Response to T. urticae

**Figure 1:**
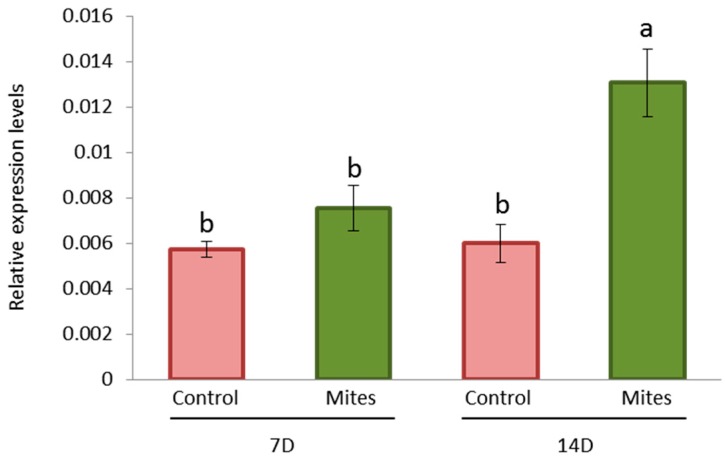
Messenger expression levels of *HvIcy6* gene in barley wild-type plants after *T. urticae* infestation, assayed by RT-qPCR. Total RNA was extracted at seven (7) or fourteen (14) days after treatment from leaves of infested or non-infested plants. Data were expressed as mRNA levels normalized to barley cyclophilin mRNA content. Different letters (a,b) indicate significant differences (*p* < 0.05, One-Way ANOVA Student Newman-Keuls test).

As HvCPI-6 had the highest in vitro inhibitory effect within the whole barley cystatin family against the CysProt of *T. urticae* [7,12], this cystatin was selected to study its expression in WT (wild-type) barley plants after *T. urticae* infestation. The results, expressed as relative expression levels, revealed that, after 14 days of *T. urticae* infestation, the expression of the *HvIcy6* gene was significantly higher in mite infested plants than in control plants (Figure 1). This result, together with previous data in our group that demonstrated that HvCPI-6 was able to confer protection against several aphids and mites pests [11,12,13], pointed us to study the in vivo effect of this cystatin in barley. For this purpose, plants overexpressing the *HvIcy6* gene were generated and analyzed.

### 2.2. Molecular Characterization of Transgenic Barley Plants Overexpressing the HvIcy6 Gene

Transgenic barley plants were generated via *Agrobacterium*-mediated transformation by ubiquitously overexpressing (OE) the *HvIcy6* gene. Homozygous plants were analyzed. Two *HvIcy6* overexpressing lines (OE Icy6: 1019 and 1082) were selected based on transgene copy number, transcript level and papain inhibitory activity for further studies (Figure S1). Following these criteria, the selected overexpressing lines showed two copies of the *HvIcy6* gene, the endogenous and the transgene, presented higher accumulation of *HvIcy6* mRNAs than control plants, and leaf protein extracts of these plants showed a higher inhibition of papain than those from non-transformed plants. In addition, the two overexpressing selected lines did not present major differences throughout the plant growth cycle in comparison to WT plants.

### 2.3. Transgenic Barley OE Icy6 Lines Show Phenotypical Differences after T. urticae Infestation

**Figure 2:**
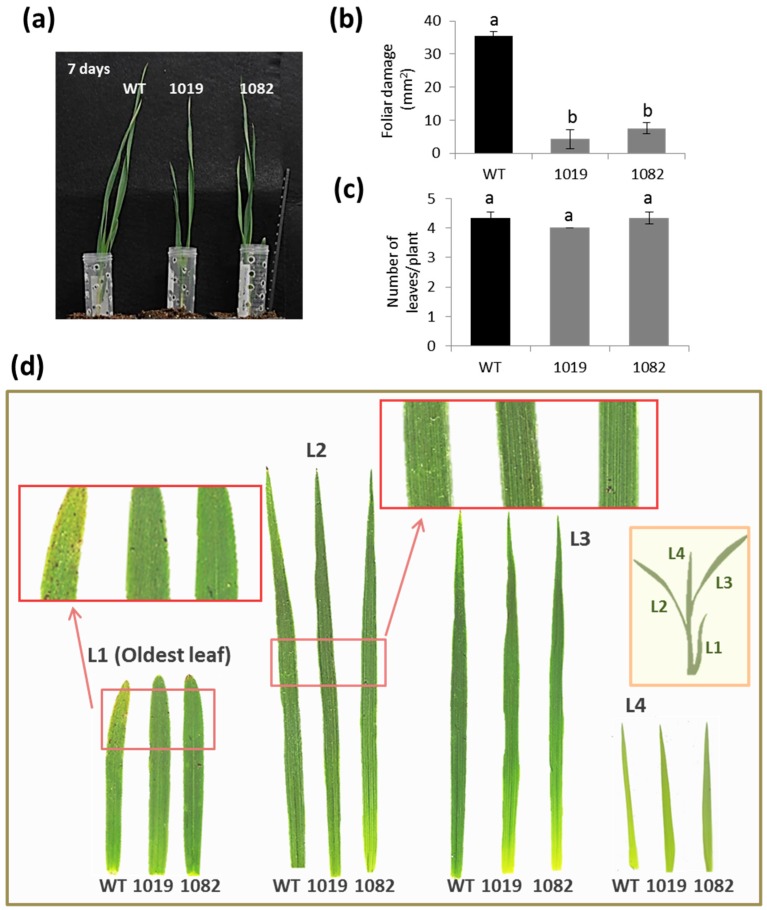
Leaf damage in wild-type and OE Icy6 lines (1019 and 1082) after 7 days of *T. urticae* infestation. (**a**) Phenotypic aspect of the plants. (**b**) Quantification of leaf feeding damage. Damage was measured as mm^2^ of injured foliar area of all leaves. (**c**) Total number of leaves per plant. Data from six independent plants. Different letters (a,b) indicate significant differences (*p* < 0.05, One-Way ANOVA Student Newman-Keuls SNK test). (**d**) Examples of leaves from transgenic and non-transgenic plants, numbered from L1 (oldest leaf) to L4 (youngest leaf) at this stage of development. Regions of L1 and L2 leaves are magnified to highlight chlorotic spots due to mite feeding.

**Figure 3:**
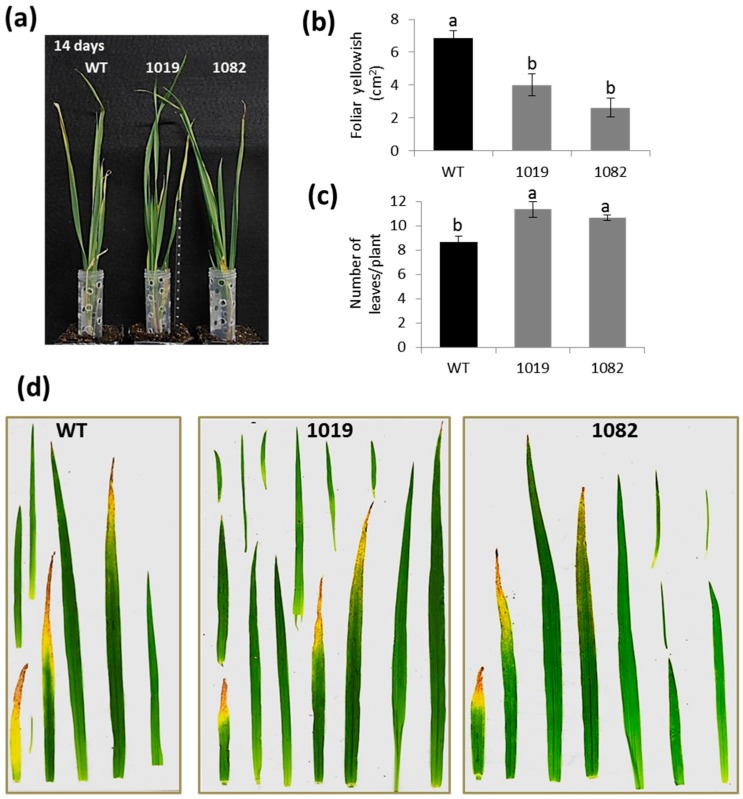
Leaf damage in wild-type and OE Icy6 lines (1019 and 1082) after 14 days of *T. urticae* infestation. (**a**) Phenotypic aspect of the plants. (**b**) Quantification of leaf yellowish measured as cm^2^ of yellow-brown foliar area of all leaves. (**c**) Total number of leaves per plant. Data from six independent plants. Different letters (**a**,**b**) indicate significant differences (*p* < 0.05, One-Way ANOVA Student Newman-Keuls SNK test). (**d**) Examples of all the leaves from individual transgenic and non-transgenic plants.

To evaluate the degree of resistance or susceptibility of modified transgenic OE Icy6 barley plants to *T. urticae*, wild-type and homozygous plants overexpressing *HvIcy6* were used to carry out in vivo experiments (Figure 2 and Figure 3). The participation of HvCPI-6 on response to *T. urticae* was first analyzed by comparing transgenic and non-transgenic plants after 7 and 14 days of mite infestation and non-infested plants. As expected, leaves from infested plants were more affected than those grown under non-infested conditions. After seven days of infestation, leaves present chlorotic spots caused by mite feeding. The worst damaged leaves were showed by WT plants, whilst the OE Icy6 lines presented only slight damages (Figure 2). At 14 days of mite infestation, the phenotypic observations were further manifested. Both, the transgenic and non-transgenic plants showed yellow spots and apical yellowish in the oldest leaves. However, this feature was more relevant in WT plants, which also had a significantly low number of leaves (Figure 3). Spider mite feeding damage was also analyzed by quantifying the leaf area injured. These results corroborated the previous phenotypical observations; the OE Icy6 transgenic lines 1019 and 1084 showed significant lower damaged foliar area than WT plants (Figure 3b). These differences were observed in both time points analyzed, at 7 and 14 days. Therefore, *T. urticae* infestation progressed faster in barley WT plants, becoming apparently more susceptible than *HvIcy6* overexpressing lines, which seem more tolerant to spider mite attack.

### 2.4. Transgenic Barley OE Icy6 Lines Affect T. urticae Performance

**Figure 4:**
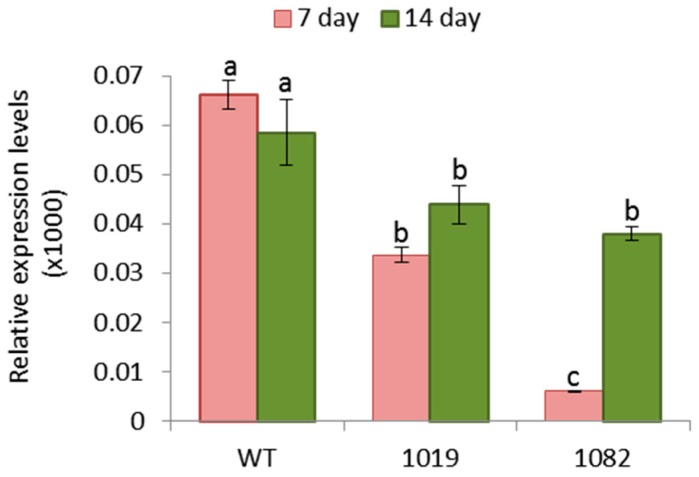
Messenger expression levels of *TuRp49* gene in barley wild-type and OE Icy6 plants after *T. urticae* infestation, assayed by RT-qPCR. Total RNA was extracted at seven or fourteen days after infestation. Data were expressed as mRNA levels normalized to barley cyclophilin mRNA content. Different letters (a,b,c) indicate significant differences (*p* < 0.05, One-Way ANOVA Student Newman-Keuls test).

To study whether the transgenic OE Icy6 barley plants are affecting *T. urticae* performance, the presence of the mite was analyzed by quantifying *T. urticae* Ribosomal Protein 49 (TuRP49) mRNA levels (Figure 4). The results showed that both, at 7 and 14 days of infestation, the mite mRNA levels were significantly higher in the WT barley plants than in OE Icy6 lines, corroborating the greater susceptibility of WT plants to *T. urticae* and the partial resistance of plants overexpressing the *HvIcy6* gene.

### 2.5. Overexpression of HvIcy6 Affects the Expression of Other Barley Genes

**Figure 5:**
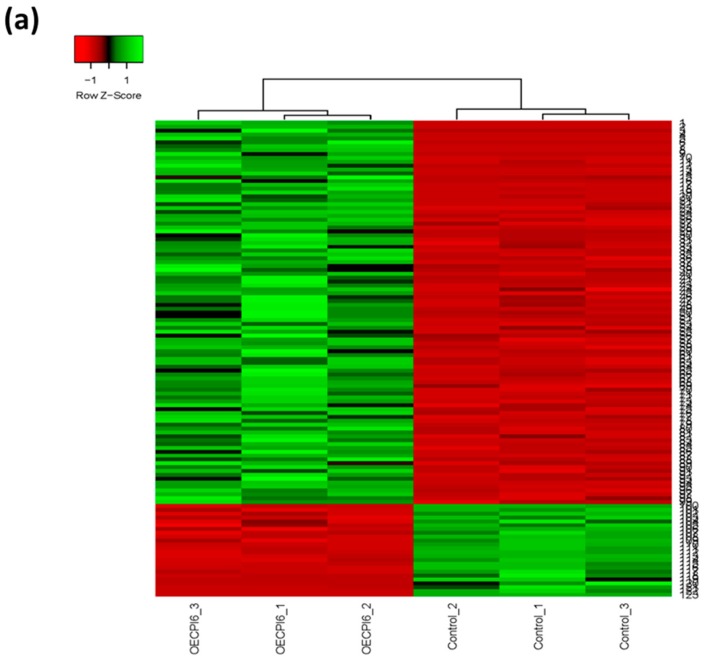

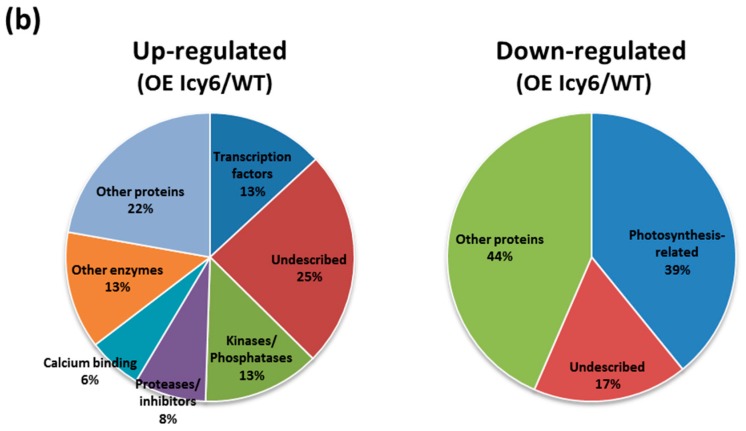
Analysis of the differentially expressed genes (DEGs) among WT and OE Icy6 plants. (**a**) Heatmap showing normalized colour intensity from the expression values of the DEGs in the three independent replicates. Green represents up-regulation and red represents down-regulation. (**b**) Circle charts showing the percentage on up- or down-regulated DEGs for the main representative categories in each subset.

To obtain more information on the molecular basis responsible for the effect of *HvIcy6* overexpression on the plant response to *T. urticae*, a RNA-seq analysis was performed in seven-day-old non-stressed plants. Only a limited number of genes were detected as differential expressed genes (DEGs), with a predominance of induced genes in the overexpression samples (99 up-regulated genes against 27 down-regulated genes, Table S1). To further analyze the expression of the DEGs, a heat map showing the differential values obtained by the three independent replicates was performed. As expected, the results of the different replicates of the same genotypic background were clustered and tended to show similar patterns (Figure 5a). Gene ontology (GO) enrichment analysis showed a reduced number of enriched GO terms (Table S2). In the up-regulated set of genes, GO terms were mainly related to regulation of gene expression by transcription factors and regulation of the biosynthesis of some macromolecules. In the down-regulated genes, transmembrane transport and magnesium binding were the main enriched GO categories. As the BARLEX definition of each gene was not reflected in the associated GO terms from the barley database, a manual categorization was performed (Figure 5b). This analysis revealed that, besides transcription factors, the percentage of other genes in the up-regulated set related to signal transduction was elevated, mainly protein kinases/phosphatases and calcium-binding proteins. In addition, the down-regulation of several genes related to the photosynthetic process was highlighted.

## 3. Discussion

Among inducible defenses, cystatins have been used for pest control against phytophagous species, such as mites, which rely on CysProt activities for the digestion of dietary proteins [12,13,15]. As herbivores have developed mechanisms through coevolution, such as changes in protease expression in the gut, to circumvent anti-digestive effects caused by the presence of an exogenous cystatin in transgenic plants [16], selection of the best natural or engineered cystatin is crucial for including cystatins in biotechnological applications [17]. Thus, the ideal cystatin should be a potent and specific inhibitor of the pest’s gut proteases and should have low inhibitory activity against plant CysProt to limit pleiotropic effects in the plant [3]. Barley HvCPI-6 protein was selected in this study since it was the strongest inhibitor of cathepsin-L- and -B-like activities of the phytophagous mites *T. urticae* and *Brevipalpus chilensis* [12], a deleterious effect on mites was previously demonstrated when it was used as a transgene [11,12,13], and our results of gene expression showed an induction of *HvIcy6* after *T. urticae* attack. However, pleiotropic effects may be found in the barley plant, since HvCPI-6 cystatin was one of the strongest inhibitor of the own barley cysteine proteases [9,18]. In this context, the aim of the present study was to assess the putative protective effect of HvCPI-6 cystatin against *T. urticae* infestation, through the analysis of transgenic barley plants overexpressing the *HvIcy6* gene, and to check changes in gene expression in these plants that could be associated to pleiotropic effects caused by the inhibition of CysProt of the plant. 

Our studies confirmed what was expected. OE Icy6 barley plants overexpressing *HvIcy6* cystatin gene remained more resistant to *T. urticae* attack when compared to wild-type plants, revealed by a significant lesser foliar damaged area, and also by lower presence of the mite determined by measuring *T. urticae* mRNA relative levels. These results were consistent with previous data from our group, since the expression of the HvCPI-6 cystatin in transgenic Arabidopsis and maize plants showed a significant development delay of *T. urticae* larvae to reach the adult stage, which was correlated with a decrease of cathepsin-L- and -B-like protease activities in mites after rearing on transgenic plants [12,13]. The prevalence and numerical importance of cysteine-protease coding sequences annotated in the genome of *T. urticae* [19], and the characterization of digestive proteases in bodies and feces of this mite [20], support that HvCPI-6 anti-mite effect in barley overexpressing plants may be mediated by inhibition of the heterologous CysProt activities involved in the digestive process of the mite. 

Transcriptomic studies have demonstrated transcriptional reprogramming upon herbivore detection to promote defense at the expense of growth [21,22], suggesting fitness costs associated with the induction of defense responses [23,24,25,26]. An enhanced primed state can ameliorate these fitness costs by optimizing the fitness benefits of rapid defense induction upon herbivore detection [27]. As an example, maize plants exposed to odor from a feeding-damaged plant resulted in enhanced expression of a trypsin inhibitor gene, which impairs armyworm performance by the inhibition of the digestion enzymes of larvae [28]. Thus, constitutively active OE Icy6 plants could be considered as an enhanced primed state that can improve the fitness costs associated with constitutive defense, while optimizing the fitness benefits of rapid defense induction upon herbivore detection. However, depending on the environmental conditions, sometimes growth must be prioritized despite herbivore attack [29,30]. In this way, recent work has questioned the paradigm of viewing costs of immunity as a trade-off. Plant growth and immunity are full integrated, sometimes as a trade-off and other times synergistically, to maximize plants fitness [31,32]. 

Herbivore attack has been shown to suppress the expression of genes encoding components of the photosynthetic pathway [21,22] suggesting that the ability to appropriately maintain photosynthesis is crucial for defense. Our transcriptomic analysis of OE Icy6 plants revealed a certain reprogramming of cellular metabolism that relies in the induction of undetermined signaling pathways and, consequently, in a lower expression of several genes related to photosynthetic activity. Thus, OE Icy6 plants would be more protected against *T. urticae* by the constitutive expression of the potent HvCPI-6 inhibitor, but, in some way, they are compromised in the rapid generation of a global defense response. As the basal expression levels of the *HvIcy6* transcript are considerably low in barley leaves, and it has a late induction pattern upon mite attack, we can speculate on the actual role of HvCPI-6 in the leaf. Whereas the outstanding inhibitory properties of this cystatin support a strong function as a defense molecule, pleiotropic inhibitory effects on barley CysProt could be deleterious for the plant. Consequently, *HvIcy6* transcript levels must be restrained, which would be in agreement with the limited differences throughout the plant growth cycle between OE Icy6 and wild-type plants. Besides, HvCPI-6 could also act as a signaling molecule to warn the plant of a serious herbivore attack. Then, the plant would readjust their metabolism carrying out a reallocation of resources to promote defense at the expense of growth.

In conclusion, the final physiological influence of genetically manipulating a protease inhibitor seems difficult to predict. Pleiotropic effects caused by its inhibitory properties should be considered before the use of these genes in biotechnological approaches to improve crop yield. In any case, the overexpression of barley protease inhibitor *HvIcy6* gene in Arabidopsis and maize plants [12,13], as well as in the barley plants analyzed in this work, have resulted potentially effective to confer protection against spider mite damage without an apparent cost in plant fitness. Therefore, whereas caution should be taken by analyzing potential deleterious pleiotropic effects, cystatins may be considered a reliable source of plant resistance genes to be used as transgenes with impact on agricultural crops by conferring enhanced levels of resistance to phytophagous pests.

## 4. Materials and Methods 

### 4.1. Plant Material

Grains of barley (*Hordeum vulgare* L. cv. “Golden Promise”) were germinated in a mixture of soil and vermiculite (3:1) and grown at 23 °C under a 16 h light/8 h darkness photoperiod for 7 days in Sanyo MLR-350-H chambers. Barley transgenic lines over-expressing the barley *HvIcy6* gene (OE Icy6) were generated in collaboration with the IPK Gatersleben, Plant Reproductive Biology Group [33]. To generate over-expression lines, the *HvIcy6* gene was transferred into the intermediate vector pUbi-AB (DNA-Cloning-Service, Hamburg, Germany) and cloned using the *Sfi*I restriction sites into the p6U binary vector (DNA-Cloning-Service, Hamburg, Germany). The *HvIcy6* gene was driven by the *Zea mays UBIQUITIN-1* promoter with first intron. Homozygous barley transgenic plants were obtained by double haploid technology [34].

### 4.2. Tetranychus urticae Infestations

A colony of the two-spotted spider mite *T. urticae* London strain (Acari: Tetranychidae), provided by Miodrag Grbic (University Western Ontario, London, Canada), was maintained on beans in a Sanyo MLR-350-H growth chamber at 25 °C under a 16 h light/8 h darkness photoperiod. This colony was transferred to barley where it was maintained under the same conditions for more than 30 generations to ensure host adaptation. To induce biotic stresses mediated by pest attack, 7-day-old barley plants, wild-type and transgenic lines overexpressing the *HvIcy6* gene, were infested with 20 barley-adapted females of *T. urticae* per plant. The infestation was performed by placing a barley leaf with the mites on the experimental plant leaves. A falcon tube with holes was used to help maintaining the leaves together. Barley plants were confined in pots with plastic cylinders covered on top by nylon nets to avoid dispersion of mites. The same isolation system was applied to control plants. Plants were further incubated at 25 °C under a 16 h light/8 h darkness photoperiod. Barley leaf damage was monitored at different time points after spider mite feeding. Leaves were harvested after 7 and 14 days of mite treatment. Samples were imaged and scanned, or frozen into liquid nitrogen and stored at −80 °C for further analysis. Leaf damage was calculated using Adobe Photoshop CS4 software (Adobe, San Jose, CA, USA). Three independent experiments were performed. 

### 4.3. Real-Time Quantitative PCR Analyses

Leaves of transgenic OE Icy6 and WT plants were used to analyze the transgene copy number and to study gene expression levels. RNA purification, cDNA synthesis and real-time reverse-transcription quantitative PCR (RT-qPCR) conditions were performed [35]. *HvIcy6* copy number was calculated by the 2^−ddCt^ method as described [35]. Quantification was standardized to barley cyclophilin (*HvCycl*) mRNA levels. Results for expression studies of the *HvIcy6* and *T. urticae* Ribosomal Protein 49 (*TuRp49*) genes are shown as relative expression levels (2^−dCt^) [36]. Expression levels of *TuRp49* were quantified by subtracting the dCt value of the barley cyclophilin from the dCt value of the mite probe to normalize for the amount of barley tissue present in each sample. Primers are specified in Table S3. 

### 4.4. Enzymatic Inhibitory Assays

The inhibitory activity of leaf protein extracts against commercial papain (Sigma-Aldrich, St. Louis, MO, USA)) was determined using the fluorogenic substrate Z-FR-AMC (*N*-carbobenzoxy-Phe-Arg-AMC, Bachem AG, Bubenddorf, Switzerland). Different extract quantities plus 10 ng of papain were incubated at room temperature in a buffer containing 100 mM sodium phosphate pH 6.0, 10 mM l-cysteine, 10 mM EDTA and 0.01% (*v*/*v*) Brij35. Then, the substrate was added and the reactions incubated for 1 h at 28 °C. Emitted fluorescence was measured with a 365 nm excitation and a 465 nm emission wavelength filter. Triplicate assays were performed for determination of each value and the average was calculated. Blanks were used to account for spontaneous breakdown of substrate and results were expressed as percentage of papain activity inhibition.

### 4.5. RNA Isolation, cDNA Library Construction and Illumina Sequencing

Total RNA was extracted from frozen barley leaves by the phenol/chloroform method, followed by precipitation with 8 M LiCl [37] and digested with DNase (Promega, Madison, WI, USA). Using poly-Toligo-attached magnetic beads, mRNAs were purified from the total RNA. Then, the mRNAs were fragmented and cDNA was synthesized using random hexamer-primers, DNA polymerase I and RNase H. The double-stranded cDNAs were purified with magnetic beads and ligated to adaptors for Illumina sequencing. The quality and quantity of the library was verified using an Agilent 2100 Bioanalyzer (Agilent, Santa Clara, CA, USA) and an ABI StepOnePlus Real-Time PCR system (Applied Biosystems, Foster City, CA, USA), respectively. The cDNA libraries were sequenced using the Illumina HiSeq2000 platform (Illumina, San Diego, CA, USA) by the Beijing Genomics Institute (BGI). More than 10 M single-end reads were obtained for each sample (three biological replicates).

### 4.6. Sequence Data Analysis

Raw reads in fastq format were first filtered, and reads with adaptor sequences and low quality reads were removed. The gene and genome sequences of *H. vulgare* retrieved from the PGSB/MIPS PlantsDB website (Available online: http://pgsb.helmholtz-muenchen.de/plant/barley/index.jsp) [38] were used as the reference databases [39]. Clean reads were pseudoaligned to the reference High Confidence genes using Kallisto RNA-seq quantification method [40]. The transcript abundance was quantified as TPM (transcripts per million) and 100 bootstrap samples were performed. Differential expressed genes (DEGs) between groups were obtained using the Wald test of the Sleuth method [41] with a b ratio (bias) higher than 1 and a q-value (false positive probability) lower than 0.01. Heat map was created using the Heatmapper web tool (Available online: http://www.heatmapper.ca/). Gene enrichment analyses were performed with the Fischer’s exact test using topGO package in R (Available online: http://bioconductor.org/packages/release/bioc/html/topGO.html) and the GO file retrieved from the PGSB/MIPS PlantsDB website (Available online: http://pgsb.helmholtz-muenchen.de/plant/plantsdb.jsp).

### 4.7. Statistical Analysis

Differences in foliar damage, number of leaves and gene expression levels among lines were assessed by One-Way ANOVA, followed by Student Newman–Keuls (SNK) multiple comparison tests. In figures, different letters indicate significant differences (SNK test, *p* < 0.05).

## Supplementary Materials

Supplementary materials can be found at http://www.mdpi.com/1422-0067/19/3/697/s1.
