# Supplementary material for: Overexpression of HvIcy6 in Barley Enhances Resistance against Tetranychus urticae and Entails Partial Transcriptomic Reprogramming
Source: Int J Mol Sci. 2018 Mar 1;19(3):697. doi: 10.3390/ijms19030697 (PMC5877558; doi:10.3390/ijms19030697)
Supplement: Supplementary file 1 [file ijms-19-00697-s001.zip › Table S2.pdf]

Table S2. List of the significant results ($p<0.01$) obtained from the enrichment gene ontology (GO) analysis of the DEGs. Only categories with more than five annotated genes are considered.

**Total DEGs (Biological process)**						
	**GO.ID**	**Term**	**Annotated**	**Significant**	**Expected**	**classicFisher**
**1**	GO:0010468	regulation of gene expression	1025	9	3.52	0.0071
**2**	GO:0060255	regulation of macromolecule metabolic process	1057	9	3.63	0.0086
**3**	GO:0019222	regulation of metabolic process	1076	9	3.69	0.0096
**Up-regulated DEGs (Biological process)**						
	**GO.ID**	**Term**	**Annotated**	**Significant**	**Expected**	**classicFisher**
**1**	GO:0019438	aromatic compound biosynthetic process	1389	9	3.66	0.008
**2**	GO:0018130	heterocycle biosynthetic process	1407	9	3.71	0.0087
**Total DEGs (Molecular function)**						
	**GO.ID**	**Term**	**Annotated**	**Significant**	**Expected**	**classicFisher**
**1**	GO:0001071	nucleic acid binding transcription factor	513	7	1.93	0.0031
**2**	GO:0003700	transcription factor activity, sequence-specific	513	7	1.93	0.0031
**3**	GO:0008378	galactosyltransferase activity	30	2	0.11	0.0057
**Up-regulated DEGs (Molecular function)**						
	**GO.ID**	**Term**	**Annotated**	**Significant**	**Expected**	**classicFisher**
**2**	GO:0001071	nucleic acid binding transcription factor	513	6	1.52	0.004
**3**	GO:0003700	transcription factor activity, sequence-specific	513	6	1.52	0.004
**Down-regulated DEGs (Molecular function)**						
	**GO.ID**	**Term**	**Annotated**	**Significant**	**Expected**	**classicFisher**
**1**	GO:0000287	magnesium ion binding	128	2	0.1	0.0045
**2**	GO:0005337	nucleoside transmembrane transporter activity	7	1	0.01	0.0055
**3**	GO:0015932	nucleobase-containing compound transmembrane	7	1	0.01	0.0055
**4**	GO:1901505	carbohydrate derivative transporter activity	12	1	0.01	0.0095
