# Supplementary material for: Overexpression of HvIcy6 in Barley Enhances Resistance against Tetranychus urticae and Entails Partial Transcriptomic Reprogramming
Source: Int J Mol Sci. 2018 Mar 1;19(3):697. doi: 10.3390/ijms19030697 (PMC5877558; doi:10.3390/ijms19030697)
Supplement: Supplementary file 1 [file ijms-19-00697-s001.zip › Table S3.pdf]

Table S3. Primer sequences used for gene amplification by RT-qPCR assays.

Gene ID	Description	Primer name	Sequence (5´ → 3´)
Hv1Hr1G004150	*HvIcy6*	Hv1Hr1G004150-F	TCAAGGACATGCCTGAAGTG
		Hv1Hr1G004150-R	CGACAGTGTCAACGAGGATG
Hv6Hr1G012570	*Cyclophilin*	Hv6Hr1G012570-F	TCCACCGGAGAGGAAGTACAGT
		Hv6Hr1G012570-R	AATGTGCTCAGAGATGCAAGGA
tetur18g03590	*TuRP49*	tetur18g03590-F	CTTCAAGCGGCATCAGAGC
		tetur18g03590-R	CGCATCTGACCCTTGAACTTC
