# Supplementary material for: Overexpression of HvIcy6 in Barley Enhances Resistance against Tetranychus urticae and Entails Partial Transcriptomic Reprogramming
Source: Int J Mol Sci. 2018 Mar 1;19(3):697. doi: 10.3390/ijms19030697 (PMC5877558; doi:10.3390/ijms19030697)
Supplement: Supplementary file 1 [file ijms-19-00697-s001.zip › Figure S1.pdf]

(a)

Construct	Event	Number of lines analysed	Selected lines
**OE Icy6**	E01	5	1019, 1048
	E02	15	1082, 1085

(b)

Line	$2^{-\Delta\Delta Ct}$ ± CV	CN
WT	1.12 ± 0.11	**1**
1019	2.12 ± 0.19	**2**
1048	6.25 ± 0.06	**6**
1082	1.81 ± 0.03	**2**
1085	1.94 ± 0.08	**2**

(c)

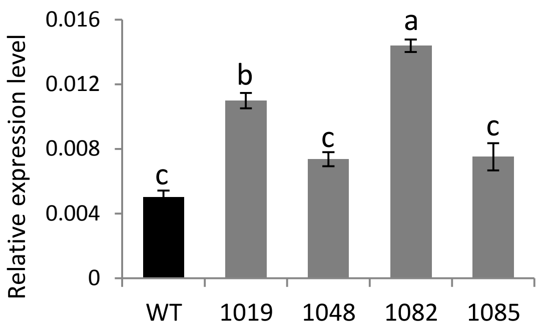

(d)

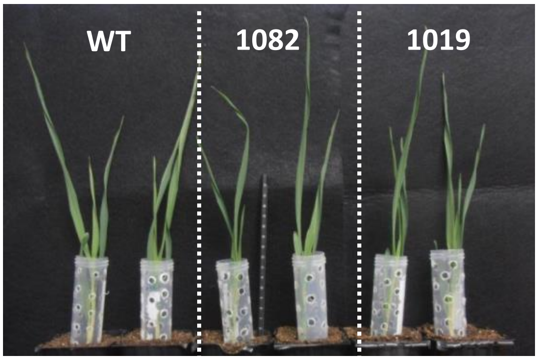

(e)

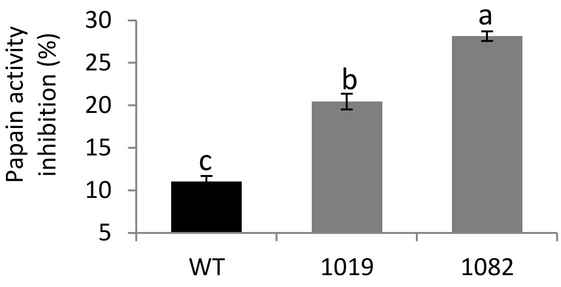

Figure S1. Characterization of overexpressing *HvIcy6* transgenic homozygous barley lines. **(a)** Number of independent homozygous lines per transformation event and selected lines used for molecular characterization. **(b)** Estimation of transgene copy number by qRT-PCR assays coupled to the $2^{-\Delta\Delta Ct}$ method. Values are expressed as the average ± coefficient of variation (CV) of triplicate measurements. *Hv4hppd* and *cyclophilin* genes were used as references for single copy and constitutive expression, respectively. CN: copy number for each group. **(c)** Relative expression levels of the *HvIcy6* gene in transgenic barley lines assessed by qRT-PCR and normalized to barley *cyclophilin* mRNA content. **(d)** Phenotypic aspect of the plants at ten days of development. **(e)** Commercial papain inhibitory capacity of leaf extracts expressed as percentage of inhibition of papain activity. Different letters indicate significant differences (P<0.05, One-Way ANOVA Student Newman-Keuls test).
