# Supplementary material for: Overexpression of HvIcy6 in Barley Enhances Resistance against Tetranychus urticae and Entails Partial Transcriptomic Reprogramming
Source: Int J Mol Sci. 2018 Mar 1;19(3):697. doi: 10.3390/ijms19030697 (PMC5877558; doi:10.3390/ijms19030697)
Supplement: Supplementary file 1 [file ijms-19-00697-s001.zip › Table S1.pdf]

Table S1. Differentially expressed genes between overexpressing *HvIcy6* and wild-type plants obtained by Kallisto/Sleuth method (qval<0.01; b>±1).

target_id	pval	qval	b	BARLEX
**HORVU3Hr1G070020.1**	3.12E-37	4.50E-34	5,75	**Cysteine proteinase inhibitor 8 (HvCPI-6)**
**HORVU4Hr1G052030.1**	2.25E-61	1.30E-57	5,47	FAR1-related sequence 5
**HORVU2Hr1G059080.1**	7.63E-11	8.51E-09	4,97	Tetratricopeptide repeat containing protein
**HORVU4Hr1G085370.1**	5.62E-22	3.33E-19	4,50	MADS-box transcription factor family protein
**HORVU6Hr1G049800.1**	4.58E-21	2.52E-18	4,49	cell division cycle 48
**HORVU3Hr1G044780.1**	4.66E-05	0.00120049	4,44	Protein transport protein SEC23-1
**HORVU7Hr1G068560.10**	3.80E-53	1.76E-49	4,42	Ubiquitin-like-specific protease 1A
**HORVU4Hr1G090490.2**	4.38E-17	1.44E-14	4,01	Ulp1 protease family, C-terminal catalytic domain containing protein
**HORVU3Hr1G043690.1**	5.87E-06	0.00020713	3,79	HEAT repeat-containing protein
**HORVU1Hr1G049390.2**	4.27E-14	8.88E-12	3,79	undescribed protein
**HORVU4Hr1G014520.1**	4.73E-06	0.00017293	3,62	unknown function
**HORVU7Hr1G109020.9**	2.20E-06	8.88E-05	3,56	Cortical cell-delineating protein
**HORVU4Hr1G041260.1**	9.37E-13	1.64E-10	3,56	undescribed protein
**HORVU4Hr1G029140.1**	0.00042663	0.00772811	3,45	Ulp1 protease family, C-terminal catalytic domain containing protein
**HORVU1Hr1G008830.2**	4.63E-05	0.00119411	3,42	unknown function
**HORVU2Hr1G033300.1**	5.01E-06	0.00018173	3,42	Ulp1 protease family, C-terminal catalytic domain containing protein
**HORVU7Hr1G093410.1**	2.38E-10	2.39E-08	3,36	unknown function
**HORVU1Hr1G008840.3**	4.76E-17	1.53E-14	3,17	unknown function
**HORVU3Hr1G117780.1**	2.51E-25	1.87E-22	3,15	Chromosome 3B, genomic scaffold, cultivar Chinese Spring
**HORVU5Hr1G041210.2**	4.11E-07	2.03E-05	3,14	receptor-like kinase 1
**HORVU2Hr1G093530.3**	0.00055329	0.00959363	3,05	Chromosome 3B, genomic scaffold, cultivar Chinese Spring
**HORVU3Hr1G044170.1**	9.54E-07	4.23E-05	3,04	Ulp1 protease family, C-terminal catalytic domain containing protein
**HORVU6Hr1G067630.1**	0.00036644	0.00679391	2,99	AAA-type ATPase family protein
**HORVU4Hr1G028400.1**	0.00023509	0.00473797	2,87	G-type lectin S-receptor-like serine/threonine-protein kinase
**HORVU2Hr1G003920.1**	5.60E-05	0.00139753	2,75	Phytosulfokine receptor 2
**HORVU0Hr1G014630.8**	1.27E-06	5.51E-05	2,72	Protein kinase superfamily protein
**HORVU6Hr1G093140.1**	6.84E-05	0.00165859	2,63	receptor-like protein kinase 1
**HORVU4Hr1G076610.1**	3.00E-10	2.94E-08	2,46	unknown function
**HORVU5Hr1G070660.1**	7.83E-05	0.00184389	2,28	undescribed protein
**HORVU1Hr1G082310.1**	7.88E-11	8.75E-09	2,22	Ethylene-responsive transcription factor 4
**HORVU0Hr1G002410.1**	2.22E-07	1.16E-05	2,16	unknown function
**HORVU0Hr1G002390.1**	0.00020488	0.0042362	2,01	unknown function
**HORVU3Hr1G056980.2**	6.34E-05	0.00155235	1,80	myb domain protein 112
**HORVU1Hr1G008900.2**	0.00032373	0.0061285	1,72	Chromosome 3B, genomic scaffold, cultivar Chinese Spring
**HORVU1Hr1G070250.2**	1.63E-14	3.85E-12	1,63	WRKY DNA-binding protein 33
**HORVU1Hr1G019410.1**	2.93E-06	0.00011318	1,58	HXXXD-type acyl-transferase family protein
**HORVU7Hr1G092530.27**	2.31E-06	9.24E-05	1,57	diacylglycerol kinase 5

**HORVU1Hr1G090720.1**	2.23E-06	8.96E-05	1,54	malate dehydrogenase
**HORVU6Hr1G080340.11**	2.45E-06	9.70E-05	1,51	Ethylene-responsive transcription factor 5
**HORVU2Hr1G093140.1**	6.56E-19	2.66E-16	1,50	alpha/beta-Hydrolases superfamily protein
**HORVU4Hr1G010770.2**	8.61E-06	0.00028735	1,50	unknown function
**HORVU3Hr1G086230.4**	3.47E-10	3.35E-08	1,50	Calcium-dependent lipid-binding (CaLB domain) family protein
**HORVU7Hr1G101790.1**	4.84E-11	5.59E-09	1,45	Calcium-binding EF-hand family protein
**HORVU7Hr1G006600.3**	0.00045122	0.00811335	1,44	Chromosome 3B, genomic scaffold, cultivar Chinese Spring
**HORVU5Hr1G113950.1**	1.83E-23	1.25E-20	1,43	DCD (Development and Cell Death) domain protein
**HORVU7Hr1G041300.1**	2.31E-05	0.00066144	1,42	Protein TIFY 11e
**HORVU4Hr1G089510.3**	3.27E-05	0.0008857	1,40	beta-amylase 5
**HORVU4Hr1G025180.1**	3.31E-06	0.00012609	1,40	Cytochrome P450 superfamily protein
**HORVU5Hr1G072770.1**	1.71E-17	5.81E-15	1,39	undescribed protein
**HORVU6Hr1G067740.3**	7.32E-07	3.34E-05	1,39	Protein NRT1/ PTR FAMILY 8.3
**HORVU5Hr1G034830.3**	7.33E-05	0.0017542	1,38	WRKY family transcription factor
**HORVU6Hr1G013700.1**	8.46E-11	9.35E-09	1,36	GDSL esterase/lipase
**HORVU5Hr1G113840.2**	6.78E-06	0.00023465	1,35	Pentatricopeptide repeat-containing protein
**HORVU4Hr1G056210.7**	1.87E-05	0.00055431	1,33	Protein phosphatase 2C family protein
**HORVU2Hr1G034280.2**	5.39E-07	2.57E-05	1,32	Lipid transfer protein
**HORVU2Hr1G024740.2**	0.00032195	0.00611495	1,31	Hexosyltransferase
**HORVU6Hr1G032750.2**	0.00034853	0.00653441	1,29	Protein SCAI
**HORVU7Hr1G082360.1**	9.09E-07	4.04E-05	1,28	UDP-Glycosyltransferase superfamily protein
**HORVU5Hr1G123970.5**	7.12E-08	4.25E-06	1,27	Beta-fructofuranosidase, insoluble isoenzyme 4
**HORVU7Hr1G101740.2**	1.30E-06	5.62E-05	1,25	Calcium-binding EF-hand family protein
**HORVU4Hr1G054770.1**	2.62E-16	7.87E-14	1,25	COBRA-like protein 7
**HORVU7Hr1G083680.1**	7.35E-05	0.00175629	1,23	undescribed protein
**HORVU0Hr1G007790.1**	7.35E-05	0.00175629	1,23	undescribed protein
**HORVU2Hr1G002840.4**	1.56E-23	1.09E-20	1,22	receptor-like protein kinase 1
**HORVU5Hr1G042030.1**	7.12E-06	0.00024469	1,22	Cytochrome P450 superfamily protein
**HORVU4Hr1G086660.1**	1.87E-10	1.93E-08	1,22	Cyclin-dependent protein kinase inhibitor SMR1
**HORVU2Hr1G123480.1**	1.59E-11	2.10E-09	1,21	CCR4-NOT transcription complex subunit 8
**HORVU1Hr1G001770.1**	5.89E-19	2.47E-16	1,20	Serine/threonine-protein kinase
**HORVU6Hr1G019870.1**	2.42E-07	1.25E-05	1,20	F-box-like
**HORVU2Hr1G043940.2**	2.77E-07	1.41E-05	1,15	Amyb5
**HORVU4Hr1G075090.1**	1.89E-14	4.33E-12	1,14	unknown function
**HORVU3Hr1G070340.3**	7.41E-16	2.11E-13	1,14	Protein-tyrosine phosphatase mitochondrial 1-like protein
**HORVU1Hr1G027700.1**	9.84E-09	6.99E-07	1,14	WRKY DNA-binding protein 50
**HORVU1Hr1G051180.1**	5.17E-05	0.00130751	1,14	S-adenosyl-L-methionine-dependent methyltransferases superfamily protein
**HORVU1Hr1G043040.1**	0.00059016	0.01011906	1,12	undescribed protein
**HORVU2Hr1G044540.1**	1.11E-11	1.58E-09	1,12	glutamate synthase 2
**HORVU4Hr1G072560.1**	1.13E-07	6.35E-06	1,11	Blue copper protein
**HORVU2Hr1G094090.1**	4.94E-09	3.86E-07	1,11	RING zinc finger protein-like
**HORVU5Hr1G061930.2**	1.02E-14	2.49E-12	1,11	Cytochrome P450 superfamily protein
**HORVU1Hr1G095160.1**	5.27E-05	0.00132358	1,10	undescribed protein
**HORVU5Hr1G108670.4**	9.03E-08	5.27E-06	1,10	Beta-1,3-galactosyltransferase 15

**HORVU1Hr1G057410.1**	3.08E-05	0.00084862	1,09	calmodulin like 23
**HORVU2Hr1G117400.1**	2.40E-06	9.53E-05	1,09	undescribed protein
**HORVU3Hr1G052250.1**	1.15E-08	8.07E-07	1,09	Chromosome 3B, genomic scaffold, cultivar Chinese Spring
**HORVU7Hr1G055330.1**	4.33E-17	1.44E-14	1,08	rRNA N-glycosidase
**HORVU7Hr1G113830.3**	8.57E-07	3.83E-05	1,08	WRKY family transcription factor
**HORVU3Hr1G056960.2**	2.11E-32	2.43E-29	1,07	Siroheme synthase
**HORVU7Hr1G005040.7**	7.70E-08	4.56E-06	1,07	ATP synthase subunit a, chloroplastic
**HORVU5Hr1G020240.2**	0.00027077	0.00528193	1,06	Cysteine proteinases superfamily protein
**HORVU5Hr1G028460.1**	4.71E-15	1.20E-12	1,06	Plant calmodulin-binding protein-related
**HORVU2Hr1G028720.4**	2.95E-06	0.0001136	1,04	Chymotrypsin inhibitor
**HORVU4Hr1G076820.1**	2.19E-15	5.81E-13	1,04	ZIM motif family protein
**HORVU5Hr1G097500.3**	3.57E-05	0.000957	1,03	Zinc finger protein 1
**HORVU2Hr1G027080.5**	5.32E-39	8.78E-36	1,03	serine/threonine protein kinase 1
**HORVU3Hr1G095240.6**	3.77E-08	2.42E-06	1,03	MADS-box transcription factor family protein
**HORVU2Hr1G034480.1**	0.00054751	0.00951494	1,03	Formin Homology 14
**HORVU7Hr1G071280.1**	1.90E-20	8.93E-18	1,01	Calcium-dependent protein kinase 2
**HORVU5Hr1G043630.1**	1.45E-05	0.00044368	1,00	undescribed protein
**HORVU5Hr1G068110.1**	1.18E-08	8.29E-07	1,00	basic helix-loop-helix (bHLH) DNA-binding superfamily protein
**HORVU2Hr1G041590.1**	6.04E-19	2.49E-16	-1,01	equilibrative nucleoside transporter 6
**HORVU4Hr1G016280.4**	1.14E-06	4.99E-05	-1,05	Dimerizations protein, putative
**HORVU0Hr1G036870.1**	1.69E-13	3.29E-11	-1,06	Ribulose bisphosphate carboxylase large chain
**HORVU6Hr1G068980.2**	0.00051113	0.00899659	-1,09	Transcription factor bHLH63
**HORVU3Hr1G078760.1**	0.00010582	0.00240077	-1,11	Cytochrome P450 superfamily protein
**HORVU7Hr1G064960.1**	0.00044899	0.0080825	-1,13	unknown function
**HORVU0Hr1G021860.4**	0.00021671	0.00445543	-1,14	Eukaryotic aspartyl protease family protein
**HORVU3Hr1G004790.1**	5.09E-05	0.00129255	-1,36	Photosystem II protein D1
**HORVU5Hr1G080300.1**	1.42E-17	4.90E-15	-1,43	Dehydration-responsive element-binding protein 1B
**HORVU0Hr1G026090.1**	9.54E-09	6.82E-07	-1,47	Ribulose bisphosphate carboxylase large chain
**HORVU6Hr1G060870.3**	7.05E-21	3.62E-18	-1,48	Reticulon-like protein
**HORVU5Hr1G011540.3**	6.34E-10	5.86E-08	-1,49	2-oxoglutarate (2OG) and Fe(II)-dependent oxygenase superfamily protein
**HORVU5Hr1G033540.1**	3.09E-08	2.01E-06	-1,58	ribulose bisphosphate carboxylase small chain 1A
**HORVU6Hr1G059800.2**	5.89E-21	3.16E-18	-1,92	Beta-1,3-galactosyltransferase 15
**HORVU0Hr1G038320.7**	1.37E-10	1.44E-08	-1,94	Prolyl endopeptidase
**HORVU3Hr1G055420.24**	9.70E-06	0.0003179	-2,21	XH/XS domain-containing protein
**HORVU4Hr1G087340.1**	1.98E-07	1.05E-05	-2,31	60 kDa jasmonate-induced protein
**HORVU2Hr1G019910.1**	1.33E-05	0.00041484	-2,62	flowering promoting factor 1
**HORVU3Hr1G079180.2**	2.05E-07	1.08E-05	-3,06	sigma factor binding protein 1
**HORVU6Hr1G062860.1**	0.00025654	0.0050924	-4,36	amino acid transporter 1
**HORVU3Hr1G114390.2**	0.00033948	0.00637969	-4,94	Pentatricopeptide repeat-containing protein
**HORVU6Hr1G018030.1**	7.67E-27	6.81E-24	-4,96	Chromosome 3B, genomic scaffold, cultivar Chinese Spring
**HORVU2Hr1G044560.1**	1.43E-61	1.10E-57	-5,55	Ferredoxin-dependent glutamate synthase 2
**HORVU5Hr1G054000.1**	2.23E-50	7.34E-47	-6,10	Chlorophyll a-b binding protein C, chloroplastic
